# SIRT7 Inhibits Adipose Tissue Browning Through Deacetylation of PPARγ2 at K382

**DOI:** 10.3390/cells15111028

**Published:** 2026-06-03

**Authors:** Avizit Das, Tatsuya Yoshizawa, Daisuke Yamada, Tomonori Tsuyama, Yoshifumi Sato, Tomoya Mizumoto, Takeshi Yoneshiro, Shingo Kajimura, Kazuya Yamagata

**Affiliations:** 1 Department of Medical Biochemistry, Faculty of Life Sciences, Kumamoto University, Kumamoto 860-8556, Japan; avizitdbmb@gmail.com (A.D.); ysato413@kumamoto-u.ac.jp (Y.S.); mizumoto-t@kumamoto-u.ac.jp (T.M.); 2 Cell Biology, Graduate School of Medical Science, Kyoto Prefectural University of Medicine, Kyoto 606-0823, Japan; dyamada@koto.kpu-m.ac.jp; 3 Center for Metabolic Regulation of Healthy Aging, Faculty of Life Sciences, Kumamoto University, Kumamoto 860-8556, Japan; t-tsuyama@kumamoto-u.ac.jp; 4 Division of Molecular Physiology and Metabolism, Tohoku University Graduate School of Medicine, Sendai 980-8575, Japan; yoneshiro.takeshi@lsbm.org; 5 Division of Metabolic Medicine, Research Center for Advanced Science and Technology (RCAST), The University of Tokyo, Tokyo 153-8904, Japan; 6 Beth Israel Deaconess Medical Center, Harvard Medical School, and Howard Hughes Medical Institute, Boston, MA 02215, USA; skajimur@bidmc.harvard.edu

**Keywords:** browning, beige adipocytes, SIRT7, PPARγ, deacetylation, PRDM16

## Abstract

**Highlights:**

**What are the main findings?**

- SIRT7 deficiency enhance beige cell differentiation in vivo (mice) and in vitro (mouse and human SVF cells).
- SIRT7-dependent deacetylation of PPARγ2 at K382 prevents interaction with the transcriptional coactivators of AT browning PRDM16 and PGC-1α.

**What are the implications of the main findings?**

- SIRT7 serves as a molecular brake on adipose tissue browning.
- SIRT7 may be a potential target for the treatment of metabolic disorders, including hypermetabolic conditions.

**Abstract:**

Adipose tissue (AT) browning is an inducible cellular phenomenon that promotes lipid oxidation to increase energy expenditure, reducing adiposity. Various transcription regulators involved in the AT browning process have been reported, but their complex molecular mechanisms remain poorly understood. Here, we explore the effects of SIRT7, one of seven mammalian sirtuins, on AT browning and elucidate the underlying mechanisms. SIRT7 deficiency increased the expression of browning genes in beige adipocytes differentiated from subcutaneous white AT (scWAT) stromal vascular fraction (SVF) cells isolated from adipocyte-specific *Sirt7* knockout (*Sirt7* AdKO) mice. The effect of SIRT7 on beige adipocyte differentiation was confirmed in *Sirt7* knockdown (KD) mouse scWAT and human supraclavicular brown AT (scBAT) SVF cell lines. Mechanistically, SIRT7 deacetylated PPARγ2 (peroxisome proliferator-activated receptor γ2) at lysine (K) 382, thereby attenuating interaction with the transcriptional coactivator PRDM16 (PR domain-containing 16). In differentiated beige adipocytes, the acetylation-mimicking mutant PPARγ2^K382Q^ had higher transcriptional activity compared with the deacetylation-mimicking mutant PPARγ2^K382R^. Furthermore, the interaction between endogenous SIRT7 and PPARγ2 decreased at the onset of beige adipocyte differentiation. Our findings reveal that SIRT7 is an important thermogenic regulator that puts the brake on AT browning by deacetylating PPARγ2.

## 1. Introduction

Adipose tissue (AT) plays an essential role in maintaining body homeostasis through metabolic adaptation, which involves processes such as lipolysis, lipogenesis, adipogenesis, and thermogenesis [1]. AT exists in two main forms: white adipose tissue (WAT) and brown adipose tissue (BAT). WAT primarily functions as an energy reservoir by storing excess calories in the form of lipids. In contrast, BAT is specialized in energy expenditure through a process known as non-shivering thermogenesis. This process is mediated by uncoupling protein 1 (UCP1), which dissipates the proton gradient without driving ATP synthesis, thereby releasing energy directly as heat [2,3]. In addition, WAT can undergo a phenotypic switch to a brown-like phenotype, a process referred to as browning, which is triggered by various environmental and physiological stimuli [3]. These inducible brown-like adipocytes, also known as brite or beige adipocytes, are a distinct type of adipocyte that develops either by de novo differentiation from progenitors within subcutaneous WAT (scWAT) or by trans-differentiation from white adipocytes [3,4]. Beige adipocyte differentiation can also be induced by cold stimulation, food, physical exercise, burn injury, sepsis, and pathological conditions such as cancer cachexia [2,3,5]. Several endogenously produced molecules, including thyroid hormones and their metabolites [6,7], fibroblast growth factor 21 (FGF21) [8], irisin [9], and lactate [10], have been shown to promote AT browning. In human, BAT gradually fades with age, and adult human BAT is more heterogenous than mouse. Interestingly, UCP1-expressing adipocytes found in the supraclavicular BAT (scBAT) depot share molecular features with beige adipocytes previously characterized in mice, rather than with classical brown adipocytes. In contrast, adipocytes located in the deep neck region exhibit molecular profiles more consistent with those of classical brown adipocytes [11]. The metabolic effects of AT browning are context-dependent. Although the activation of AT browning has been widely recognized as a potential therapeutic strategy for obesity and metabolic disorders, excessive activation may have adverse effects, particularly under hypermetabolic conditions [12]. Therefore, it is important to understand the mechanisms regulating both the activation and suppression of the AT browning process.

Many studies have identified positive and negative regulators of beige adipocyte differentiation. Most of the signaling pathways identified regulate three core transcription factors: PRDM16 (PR domain-containing 16), PPARγ (peroxisome proliferator-activated receptor γ), and PGC-1α (encoded by the *Ppargc1a* [PPARγ coactivator 1-alpha] gene) [13]. PPARγ exists in two isoforms, PPARγ1 and PPARγ2, which differ structurally in that PPARγ2 contains an additional 30 amino acids at its N-terminal domain. Moreover, whereas PPARγ1 is broadly expressed across multiple tissues, PPARγ2 expression is largely restricted to AT [14]. The transcriptional activity of PPARγ is further regulated through various post-translational modifications, including phosphorylation, SUMOylation, ubiquitylation, GlcNAcylation, and acetylation, each of which can modulate its functional output in a context-dependent manner [15]. Specific post-translational modifications in PPARγ have been suggested to control specific subsets of PPARγ target genes [15]. For instance, during AT browning, deacetylation of PPARγ on lysine (K) 268 and 293 facilitates its interaction with the thermogenic coactivator PRDM16. This interaction promotes the preferential activation of BAT-associated gene programs while suppressing gene expression patterns characteristic of visceral adipose tissue [16].

Sirtuins (SIRT1–7 in mammals) are nicotinamide adenine dinucleotide (NAD^+^)-dependent lysine deacetylases/deacylases that regulate diverse biological processes, including energy metabolism, stress resistance, tumorigenesis, and aging. While several sirtuin family members have been shown to facilitate AT browning [16,17,18], we recently revealed that SIRT7 exerts an opposing inhibitory effect [19]. We observed that the expression of browning-related genes was significantly elevated in the inguinal WAT of norepinephrine-treated adipocyte-specific *Sirt7* knockout (*Sirt7* AdKO) mice compared with control mice. However, the underlying mechanisms were unclear. Additionally, we previously reported that SIRT7 deacetylates PPARγ2 at lysine residue 382 (K382), a modification associated with enhanced lipogenesis in C3H10T1/2-derived white adipocytes [20]. These results provided insight into the roles and underlying mechanisms of SIRT7-mediated PPARγ2 deacetylation at K382 in AT browning.

In the present study, conducted to elucidate the precise role of SIRT7 in AT browning, we demonstrate that SIRT7 deficiency enhances beige adipocyte differentiation from mouse scWAT and human scBAT stromal vascular fraction (SVF) cells. SIRT7-dependent deacetylation of PPARγ2 at K382 prevented complex formation with PRDM16 and PGC-1α. Our results establish SIRT7 as a critical thermogenic suppressor that puts the brake on AT browning.

## 2. Materials and Methods

### 2.1. Animals

*Adipoq*-Cre mice (JAX 010803), backcrossed for more than eight generations onto the C57BL/6J genetic background, were generously provided by Dr. Evan D. Rosen [21] and were intercrossed with *Sirt7* floxed mice (*Sirt7*^fl/fl^) [19] to generate *Sirt7* AdKO (*Sirt7*^fl/fl^; *Adipoq*-Cre^+/−^) mice and *Sirt7*^+/+^; *Adipoq*-Cre^+/−^ littermates were used as a control for this study. For the in vivo experiment, 10-week-old male control and *Sirt7* AdKO mice were housed in separate cages (*n* = 3 per group) and administered norepinephrine daily at a dose of 1 mg/kg body weight for five consecutive days to induce AT browning, as previously reported [19].

All experimental procedures were approved by the Kumamoto University Ethics Review Committee for Animal Experimentation (Approval ID: A2023-027R1, from 28 June 2023, A2025-043 from 17 April 2025) and were conducted in accordance with the ARRIVE guidelines. Mice were group-housed at a maximum of five animals per cage under a 12 h light/dark cycle in a specific pathogen-free, temperature-controlled facility maintained at 22–23 °C with 40–80% humidity. Animals had ad libitum access to water and a standard chow diet (CE-2, CLEA Japan Inc., Tokyo, Japan). Group allocation was determined solely based on genotype; neither randomization nor blinding procedures were applied. The health status of the mice was monitored at least once daily by both the research team and veterinary staff. Humane end-points included ≥25% body weight loss, persistent recumbency/crouching, respiratory abnormalities, severe diarrhea, generalized paralysis, or circling. No animals reached these endpoints, and no adverse events requiring intervention were observed during the study period. Consequently, no separate exclusion table is provided.

### 2.2. Cell Culture

The procedure for isolating SVF cells from scWAT has been described previously [19,22]. Briefly, inguinal WAT collected from 6–8-week-old male control (*Sirt7*^+/+^; *Adipoq*-Cre^+/−^) and *Sirt7* AdKO mice was finely minced and digested with collagenase and dispase II at 37 °C for 50 min under continuous shaking. The digested tissue suspension was then filtered and centrifuged at 700× *g* for 5 min. The resulting cell pellet was resuspended in growth medium consisting of Dulbecco’s Modified Eagle’s Medium (DMEM) (high glucose with L-glutamine and phenol red and without sodium pyruvate; Fujifilm Wako Pure Chemical Corp., Osaka, Japan) supplemented with 10% fetal bovine serum (FBS) (Biosera, Nuaillé, France) and 100 U/mL penicillin/streptomycin (Meiji Seika Pharma Co., Ltd., Tokyo, Japan), and plated onto cell culture dishes.

Mouse scWAT SVF cell lines [23] were cultured in the same medium. For differentiation into beige adipocytes, cells at 95–97% confluence (confluent but not too packed) were incubated with differentiation medium consisting of growth medium supplemented with 500 μM isobutylmethylxanthine (Sigma-Aldrich, St. Louis, MI, USA), 125 μM indomethacin (Sigma-Aldrich), 2 μg/mL dexamethasone (Sigma-Aldrich), 5 μg/mL insulin (Fujifilm Wako Pure Chemical Corp.), 1 nM T3 (Sigma-Aldrich), and 0.5 μM rosiglitazone (Sigma-Aldrich). We carefully monitored cell confluency to ensure that the cultures did not become overconfluent and that there were no variations in cell density between individual samples/groups. After 2 days of induction, the medium was replaced with growth medium containing 5 μg/mL insulin, 1 nM T3, and 0.5 μM rosiglitazone, and cells were further cultured until adipocyte differentiation became apparent. To confirm beige adipocyte differentiation, the gene expressions of white/beige (*Hoxc9*) and brown (*Zic1* and *Meox1*) adipocyte markers [24,25] were examined by quantitative polymerase chain reaction (qPCR) (Figure S1).

Human scBAT SVF cell lines (the original stock before single cloning of clone #11-1) [11] were maintained in MesenCult-ACF Medium (Stemcell Technologies, Vancouver, BC, Canada) supplemented with 2 mM L-glutamine (Stemcell Technologies) and 100 U/mL penicillin/streptomycin on type I collagen plates. For differentiation into beige adipocytes, MesenCult Adipogenic Differentiation Medium (Human) (Stemcell Technologies) was used according to the manufacturer’s protocol.

Mouse BAT SVF cell lines [26] were maintained in growth medium consisting of DMEM/Nutrient F-12 [1:1] (Gibco BRL, Grand Island, NY, USA) supplemented with 10% FBS (Biosera) and 100 U/mL penicillin/streptomycin. For differentiation into brown adipocytes, the cells were treated for 2 days with 125 mM indomethacin, 2 mg/mL dexamethasone, and 0.5 mM isobutylmethylxanthine in maintenance medium (growth medium supplemented with 1 nM T3 and 85 nM insulin). Following the 2-day induction period, cells were maintained in maintenance medium, which was refreshed every other day until the appearance of intracellular lipid droplets. To confirm the appropriate differentiation to brown adipocytes, the gene expressions of white/beige (*Hoxc9*) and brown (*Zic1* and *Meox1*) adipocyte markers [24,25] were examined by qPCR (Figure S1).

LentiX293T cells (Clontech Laboratories, Mountain View, CA, USA) were maintained in DMEM (high glucose with 1 mM sodium pyruvate; Fujifilm Wako Pure Chemical Corp.) containing 10% FBS (Biosera).

### 2.3. Oil Red O Staining

Oil Red O solution (0.3% *v*/*v*; Sigma-Aldrich) was prepared in isopropanol, diluted with water at a 3:2 ratio, and passed through a 0.22 μm filter to remove particulates prior to use. For lipid staining, cells were fixed in 10% neutral buffered formalin, rinsed with PBS, and briefly treated with 60% isopropanol. Cells were then incubated with the filtered Oil Red O working solution to stain intracellular lipid droplets. Following incubation, cells were washed sequentially with 60% isopropanol and PBS to remove excess stain. Lipid droplets were visualized using a BZX-700 microscopy system (Keyence, Inc., Osaka, Japan), and the ratio of lipid area to total area was quantified using a BZ-X Analyzer (Keyence, Inc.).

### 2.4. Gene Expression Analysis

**Figure 1:**
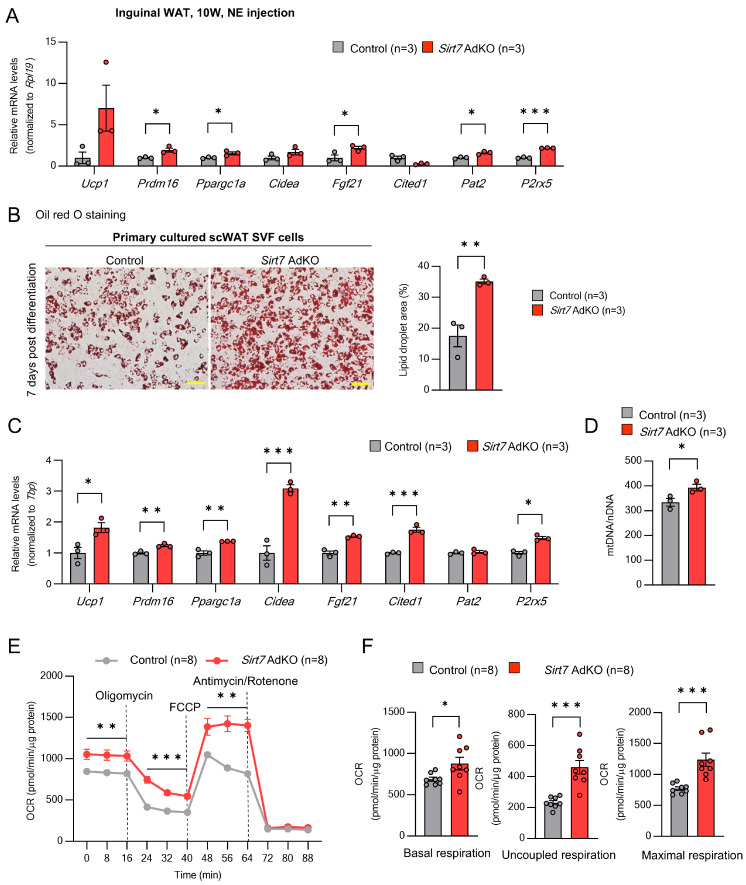
SIRT7 deficiency enhances beige adipocyte differentiation from primary cultured scWAT SVF cells of *Sirt7* AdKO mice. (**A**) Real-time qPCR analysis of browning marker genes in inguinal WAT of control (*Sirt7*^+/+^; *Adipoq*-Cre^+/−^) and *Sirt7* AdKO (*Sirt7*^fl/fl^; *Adipoq*-Cre^+/−^) mice treated with norepinephrine. (**B**) Oil red O staining of fully differentiated beige adipocytes (7 days post-differentiation) derived from cultured scWAT SVF cells isolated from control and *Sirt7* AdKO mice (left panel, scale bar = 100 μm), with quantification of lipid droplet area (right panel). (**C**) Expressions of browning marker genes analyzed by real-time qPCR in the differentiated cells described in (**B**). (**D**) Measurement of mtDNA copy number in the cells described in (**B**). (**E**,**F**) Mitochondrial stress test in the cells described in (**B**). OCR recorded over time (**E**) and quantification of mitochondrial respiration parameters (**F**). Data are presented as mean ± SEM. For (**B**–**D**), scWAT SVF cells were isolated from three mice per group. For (**E**,**F**), scWAT SVF cells were isolated from one mouse per group and independently cultured and differentiated into eight wells per group. * *p* < 0.05, ** *p* < 0.01, *** *p* < 0.001 with a two-tailed Student’s *t*-test.

Total RNA was isolated using Sepasol RNA I Super reagent (Nacalai Tesque, Inc., Kyoto, Japan), and RNA purity and quantity were assessed using a NanoDrop spectrophotometer (Thermo Scientific, Waltham, MA, USA). One microgram of total RNA (with acceptable 260/280 and 260/230 ratios) from each sample was used for cDNA synthesis using a PrimeScript RT reagent kit (Takara Bio, Inc., Shiga, Japan). Real-time quantitative PCR (qPCR) was performed using SYBR Premix Ex Taq II (Takara) on an applied Biosystems ViiA7 real-time PCR system (Thermo Fisher Scientific, Foster City, CA, USA). Relative gene expression levels were normalized to ribosomal protein L19 (*Rpl19*) for Figure 1A or to TATA box-binding protein (*Tbp*) for other figures. Melt curve analysis was used to confirm the amplification specificity of the primers. Primer sequences are listed in Supplementary Table S1.

### 2.5. Western Blotting

Whole-cell lysates were prepared by resuspending cells in RIPA buffer (50 mM Tris-HCl pH 8.0, 150 mM NaCl, 0.1% SDS, 1% NP-40, 5 mM EDTA, and 0.5% sodium deoxycholate) supplemented with a protease inhibitor cocktail (Nacalai Tesque). Proteins were resolved by SDS-PAGE and transferred onto Immobilon-P PVDF membranes (Merck Millipore, Burlington, MA, USA). Membranes were then incubated with the following primary antibodies: anti-SIRT7 (clone D3K5A, #5360; Cell Signaling Technology, Danvers, MA, USA), anti-β-actin (#177-3; MBL, Woburn, MA, USA), anti-HA (clone 3F10, #11867423001; Roche Applied Science, Penzberg, Germany), anti-DYKDDDK (FLAG) tag (clone 1E6, #018-22381; Wako Pure Chemical Industries, Osaka, Japan), anti-PPARγ (#16643-1-AP; Proteintech Group, Inc., Rosemont, IL, USA), and anti-acetylated lysine (#9441; Cell Signaling Technology). Following incubation with the appropriate secondary antibodies, immunoreactive bands were detected using Chemis-Lumi One Super reagent (Nacalai Tesque, Inc.) and a ChemiDoc Imaging System (Bio-Rad Laboratories, Hercules, CA, USA). Band intensities were quantified using Image Lab software version 6.0 (Bio-Rad).

### 2.6. Evaluation of Mitochondrial DNA Copy Number

Cells were lysed for approximately 12–18 h at 55 °C in DNA extraction buffer (100 mM Tris-HCl pH 8.5, 5 mM EDTA, 0.2% SDS, 200 mM NaCl) containing 300 μg/mL proteinase K (Nacalai Tesque). Following genomic DNA extraction, mitochondrial DNA (mtDNA) copy number was expressed as the ratio of mtDNA to nuclear DNA (nDNA). mtDNA; NADH dehydrogenase 1, mitochondrial (*mt-Nd1*), nDNA; platelet/endothelial cell adhesion molecule 1 (*Pecam1*). The primer sequences are listed in Supplementary Table S1.

### 2.7. Extracellular Flux Measurement

The oxygen consumption rate (OCR) was measured using a Seahorse XF24 Extracellular Flux Analyzer (Seahorse Bioscience, Santa Clara, CA, USA). Beige adipocytes differentiated from SVF cells cultured in a 24-well XF24 microplate (Seahorse Bioscience), and the OCR was measured at 37 °C according to the manufacturer’s instructions. Prior to measurement, cells were equilibrated for 30 min in pre-warmed, CO_2_ free assay medium (Agilent Seahorse XF base medium [Agilent Technologies, Inc., Santa Clara, CA, USA] supplemented with 25 mM glucose, 1 mM sodium pyruvate, and 2 mM L-glutamine). A mitochondrial stress test was then performed by sequential injection of 5 μM oligomycin, 10 μM FCCP, and 0.5 μM rotenone/antimycin A (Agilent Technologies, Inc.) to assess uncoupled respiration, maximal respiratory capacity, and non-mitochondrial respiration, respectively. The OCR values were normalized to total protein content, and the results are reported as pmol of O_2_/min/μg protein.

### 2.8. Plasmid Construction

pSIREN-RetroQ (Clontech), pRSV-Rev (REV) (#12253; Addgene, Watertown, MA, USA), pMDLg/pRRE (RRE) (#12251; Addgene), pMD2.G (VSVG) (#12259; Addgene), pLKO.1-blast (#26655; Addgene), and pRL-TK (Promega, Madison, WI, USA) were obtained from the indicated suppliers. pSIREN-RetroQ-mSirt7 (target sequence: 5′-CGGGATACCATTGTGCACTTT-3′), pcDNA3.1-3 × HA-PPARγ2^K382R^, pcDNA3.1-3 × HA-PPARγ2^K382Q^, pcDNA3.1-FLAG-mPRDM16, pcDNA3-FLAG-mPGC-1α, pcDNA3.1-FLAG-mSIRT1, and pUC-3 × PPRE-tk-LUC were generated as previously described [20,27,28,29,30]. To generate pMXs-Puro-HA-mPPARγ2^K382R^ and pMXs-Puro-HA-mPPARγ2^K382Q^, the PacI/XhoI fragment of pcDNA3.1-HA-PPARγ2^K382R^ and pcDNA3.1-HA-PPARγ2^K382Q^ was inserted into the pMXs-Puro plasmid. For knockdown (KD) of human *Sirt7*, oligonucleotides encoding human *Sirt7* shRNA (target sequence: 5′-ATGAAAAAGTGTGAACTTTAT-3′) were cloned into pLKO.1-blast.

### 2.9. Retroviral and Lentiviral Transduction

Retroviral vectors for mouse *Sirt7* KD (pSIREN-RetroQ-mSirt7, with pSIREN-RetroQ as negative control) and for overexpression of mouse PPARγ2 mutants (pMXs-Puro-HA-mPPARγ2^K382R^ and pMXs-Puro-HA-mPPARγ2^K382Q^) were transfected into Plat-E packaging cells using jetPRIME transfection reagent (Polyplus, Illkirch-Graffenstaden, France). Virus-containing supernatants were collected 48 h post-transfection and passed through a 0.2 μm syringe filter. Cells were then infected with the filtered retroviral supernatant for 8 h, followed by selection with 3 μg/mL puromycin for 72 h to establish stable cell lines.

Lentiviral vectors for human SIRT7 KD (pLKO.1-blast-hSirt7, with pLKO.1-blast-control as negative control) were co-transfected with the packaging plasmids pMDLg/pRRE, pRSV-Rev, and pMD2.G into LentiX293T cells using PEI-MAX reagent (Polysciences, Warrington, PA, USA). Virus-containing supernatant was collected 48 h post-transfection and passed through a 0.45 μm syringe filter. Cells were then infected with the filtered lentiviral supernatant for 24 h, followed by selection with 1 μg/mL blasticidin (Nacalai Tesque) for 5 days to establish stable knockdown cell lines.

### 2.10. RNA-Seq Analysis

Total RNA was isolated with the RNeasy Micro Kit (74004; Qiagen, Hilden, Gemany) according to the manufacturer’s protocol. RNA-seq libraries were generated using a NEBNext Ultra RNA Library Prep Kit for Illumina (New England Biolabs, Ipswich, MA, USA), and sequenced on an Illumina NovaSeq X Plus platform using 150-bp paired-end reads. Raw reads were processed using TrimGalore (v0.6.7) to remove Illumina adapter sequences [31], followed by alignment to the mouse reference genome (GRCm39) using HISAT2 (ver. 2.2.1). The aligned reads were sorted and converted to a binary alignment/map format with SAMtools (ver. 1.14). Transcript assembly and gene quantification were performed using StringTie (ver. 2.2.1), and gene-level count matrices were generated using the Python (ver. 3) script prepDE.py3 [32]. Differentially expressed genes (DEGs) were determined using DESeq2 (ver. 1.38.0). DEGs with an adjusted *p*-value < 0.05 and an absolute fold change > 2 were subjected to Gene Ontology (GO) analysis with DAVID (ver. 6.8) [33]. The RNA-seq datasets generated in this study have been deposited in the Gene Expression Omnibus (GEO) under accession number: GSE317859.

### 2.11. Co-Immunoprecipitation Assay

LentiX293T cells were transfected with the indicated expression plasmids using jetPRIME transfection reagent, followed by overnight treatment with 1 μM rosiglitazone. Cells were then lysed in ice-cold lysis buffer (20 mM Tris-HCl pH 7.4, 200 mM NaCl, 2.5 mM MgCl_2_, 0.5% NP-40, 1 μM rosiglitazone, 1 mM PMSF, and protease inhibitor cocktail), and mechanically disrupted by passing through a 29-G needle six times. Lysates were centrifuged at 14,000× *g* for 10 min at 4 °C to remove debris, and the supernatant was collected. For co-immunoprecipitation of tagged proteins, the lysate supernatant was incubated for approximately 12–16 h at 4 °C with anti-HA tag antibody-conjugated beads (clone 4B2; Wako Pure Chemical Industries) under end-over-end mixing. To assess interactions between endogenous PPARγ and SIRT7, cell lysates were immunoprecipitated overnight at 4 °C using anti-PPARγ antibody-crosslinked resin prepared with a Pierce Crosslink Immunoprecipitation Kit (Thermo Fisher Scientific). Following five washes with lysis buffer, bound proteins were eluted with the kit-supplied elution buffer (pH 2.8, containing primary amine) and analyzed by Western blotting with appropriate antibody.

### 2.12. Luciferase Assay

Mouse scWAT SVF cell lines overexpressing PPARγ2^K382R^ or PPARγ2^K382Q^, as well as control or *Sirt7* KD mouse scWAT SVF cell lines, were co-transfected with a PPRE-driven firefly luciferase reporter plasmid (pUC-3 × PPRE-tk-LUC) and an internal control vector (pRL-TK) using jetPRIME transfection reagent at both the undifferentiated and differentiated stages. At 24 h post-transfection, cells were lysed in kit supplied lysis buffer, and luciferase activity was measured using the Dual-Luciferase Reporter Assay System (Promega) with firefly and Renilla luciferase substrates.

### 2.13. Acetylation Assay

Mouse scWAT SVF cell lines differentiated into beige adipocytes were harvested at 24 h and 48 h post-differentiation and mechanically disrupted by sonication (Sonifier-150; Branson Ultrasonics, Danbury, CT, USA) at 4 °C in lysis buffer supplemented with 10 mM nicotinamide and 1 μM TSA. Lysates were clarified by centrifugation at 14,000× *g* for 10 min at 4 °C and then immunoprecipitated overnight at 4 °C using anti-PPARγ antibody-crosslinked resin (described above). Immunoprecipitated proteins were eluted with the elution buffer supplied in the Pierce Crosslink Immunoprecipitation Kit. Lysine acetylation was assessed by Western blotting using an anti-acetyl lysine antibody (Cell Signaling Technology).

### 2.14. Statistical Analysis

No statistical methods were used to predetermine the sample size, and no exclusion/inclusion criteria were applied to the mice used in this study. Statistical analyses were performed using GraphPad Prism 9 (GraphPad Software). Data are expressed as mean ± standard error of the mean (SEM). Statistical comparisons between two groups were conducted using a two-tailed Student’s *t*-test. The use of parametric analysis (two-tailed Student’s *t*-test) with limited sample sizes was considered appropriate because the experimental variables assessed in this study have previously been reported to follow an approximately normal distribution in established assay systems [30]. In all analyses, *p* < 0.05 was considered to indicate a significant difference.

## 3. Results

### 3.1. SIRT7 Deficiency Enhances Beige Adipocyte Differentiation from scWAT SVF Cells of Sirt7 AdKO Mice

We previously reported that SIRT7 inhibits energy expenditure and thermogenesis through the regulation of classical BAT functions [19]. In that study, the browning phenotype was not evident at room temperature (23 °C); therefore, mice were administered norepinephrine over a five-day period to activate AT browning. Under these conditions, *Sirt7* AdKO mice exhibited upregulation of browning-associated gene programs in inguinal WAT compared with controls. Reanalysis of the data confirmed this finding with other genes (Figure 1A). Since adipose tissue browning is influenced by interactions among multiple organs and cell types, we investigated whether the enhanced AT browning observed in *Sirt7* AdKO mice occurs via a cell-autonomous mechanism. To this end, we harvested SVF cells from scWAT of *Sirt7* AdKO and control mice. As shown in Figure 1B, beige adipocytes differentiated from scWAT SVF cells of *Sirt7* AdKO mice had more lipid droplets than those from control mice. The expression of browning marker genes was also significantly upregulated in *Sirt7* KO beige adipocytes (Figure 1C). Because mtDNA content was elevated in *Sirt7* KO beige adipocytes compared with control cells (Figure 1D), we subsequently assessed mitochondrial respiration using a Seahorse XF24 Flux Analyzer with sequential treatment of oligomycin, FCCP, and antimycin A/rotenone (Figure 1E). *Sirt7* AdKO beige adipocytes showed increased basal, uncoupled, and maximal mitochondrial respiration relative to control cells (Figure 1F). Collectively, these findings indicate that SIRT7 negatively regulates mitochondrial oxidative respiration and energy expenditure by suppressing the differentiation of beige adipocyte precursors.

### 3.2. SIRT7 Deficiency Enhances Beige Adipocyte Differentiation of Mouse scWAT and Human scBAT SVF Cell Lines

**Figure 2:**
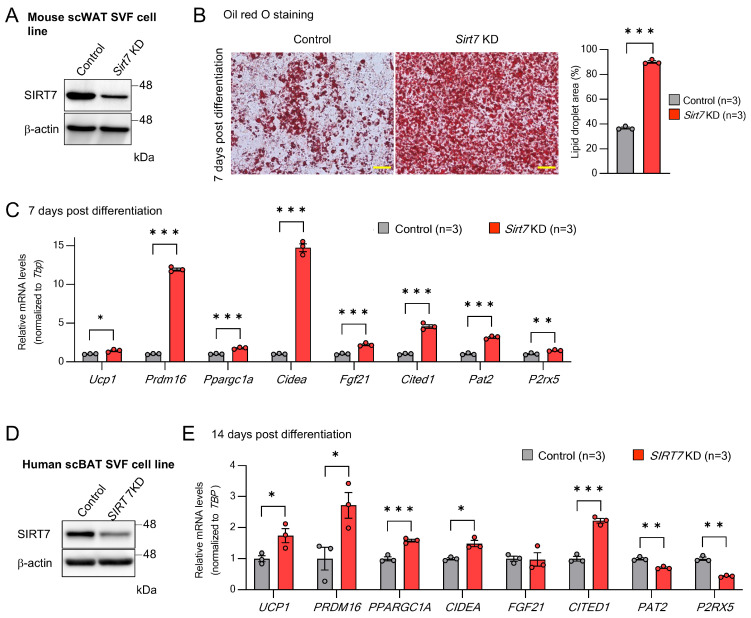
SIRT7 deficiency enhances beige adipocyte differentiation of mouse scWAT and human scBAT SVF cell lines. (**A**) Western blot analysis of SIRT7 in *Sirt7* KD mouse scWAT SVF cell lines. (**B**) Oil red O staining of fully differentiated (7 days post-differentiation) beige adipocytes from control and *Sirt7* KD mouse scWAT SVF cell lines (left panel, scale bar = 100 μm), and quantification of the stained oil red O area (right panel). (**C**) Real-time qPCR analysis of browning marker genes in the cells described in (**B**). (**D**) Western blot analysis of SIRT7 in *SIRT7* KD human scBAT SVF cell lines. (**E**) Realtime qPCR analysis of browning marker genes in fully differentiated (14 days post-differentiation) beige adipocytes from control and *SIRT7* KD human scBAT SVF cell lines. Data are presented as mean ± SEM of triplicates. * *p* < 0.05, ** *p* < 0.01, *** *p* < 0.001 with a two-tailed Student’s *t*-test.

Because primary cultures of scWAT SVF cells contain a heterogeneous cell population, the fraction of beige adipocyte precursor cells in scWAT SVF cells may differ between control and *Sirt7* AdKO mice. Therefore, we next used immortalized SVF cell lines to analyze the effect of SIRT7 deficiency on AT browning. SIRT7 expression was successfully knocked down in mouse scWAT SVF cell lines by using a retroviral system expressing shRNA targeting *Sirt7* (Figure 2A). When mouse scWAT SVF cell lines were cultured in beige adipocyte differentiation medium, lipid droplet deposition was substantially increased in *Sirt7* KD cells compared to control cells (Figure 2B). In addition, the expression levels of browning marker genes were significantly upregulated in differentiated *Sirt7* KD cells (Figure 2C). These results demonstrate that SIRT7 suppresses beige adipocyte differentiation via a cell-autonomous mechanism.

The molecular signatures of adult human brown adipocytes from scBAT, which is the most consistently found and well-studied human BAT depot, resemble those of mouse beige adipocytes. Therefore, we next examined the applicability of our findings to humans by using human scBAT SVF cell lines. The phenotypes of beige adipocytes differentiated from *SIRT7* KD human scBAT SVF cell lines regarding browning marker gene expression were almost identical to those of *Sirt7* KD mouse scWAT SVF cell lines, while lipid droplet deposition was not changed (Figure 2D,E and Figure S2). Taken together, our findings suggest SIRT7 as a critical thermogenic suppressor that puts the brake on AT browning in mice and humans.

### 3.3. Acetylation of PPARγ2 at K382 Enhances Beige Adipocyte Differentiation

**Figure 3:**
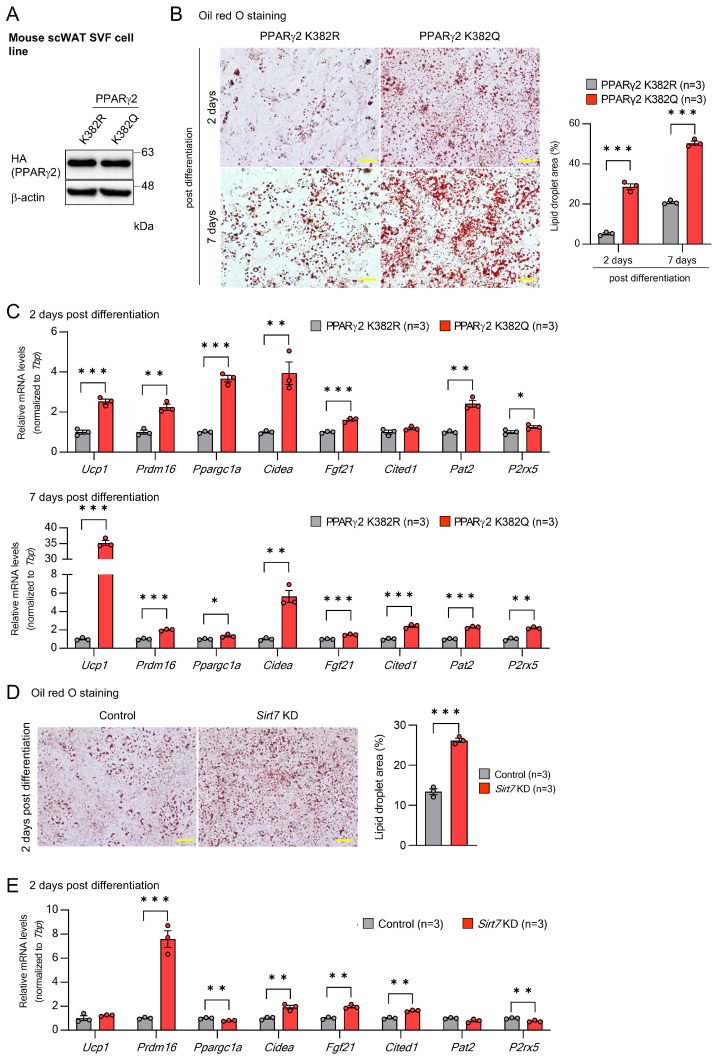
Acetylation of PPARγ2 at K382 enhances beige adipocyte differentiation. (**A**) Western blot analysis of PPARγ in PPARγ2^K382Q^- and PPARγ2^K382R^-overexpressing mouse scWAT SVF cell lines. (**B**) Oil red O staining of early differentiated (2 days post-differentiation) and fully differentiated (7 days post-differentiation) beige adipocytes from PPARγ2^K382Q^- and PPARγ2^K382R^-overexpressing mouse scWAT SVF cell lines (left panel, scale bar = 100 μm), and quantification of the stained oil red O area (right panel). (**C**) Real-time qPCR analysis of browning marker genes in the cells described in (**B**). (**D**) Oil red O staining of early differentiated (2 days post-differentiation) beige adipocytes from control and *Sirt7* KD mouse scWAT SVF cell lines (left panel, scale bar = 100 μm), and quantification of the stained oil red O area (right panel). (**E**) Real-time qPCR analysis of browning marker genes in the cells described in (**D**). Data are presented as mean ± SEM of triplicates. * *p* < 0.05, ** *p* < 0.01, *** *p* < 0.001 with a two-tailed Student’s *t*-test.

We previously reported that SIRT7 deacetylates PPARγ2 at K382, thereby enhancing lipogenesis in white adipocytes [20]. Accordingly, we assessed the contribution of PPARγ2 acetylation at K382 to beige adipocyte differentiation by using the acetylation-mimicking mutant PPARγ2^K382Q^ and the deacetylation-mimicking mutant PPARγ2^K382R^. Both PPARγ2 mutants were largely similarly overexpressed in mouse scWAT SVF cell lines (Figure 3A). In contrast to our previous findings [16], beige adipocytes differentiated from PPARγ2^K382Q^-overexpressing cells had significantly more lipid droplets than those differentiated from PPARγ2^K382R^-overexpressing cells, even at 2 days post-differentiation (Figure 3B). The expression levels of browning marker genes were already strikingly upregulated in PPARγ2^K382Q^-overexpressing cells at 2 days post-differentiation and were even higher at 7 days post-differentiation (Figure 3C). Consistently, in *Sirt7* KD mouse scWAT SVF cell lines, lipid droplet deposition and browning marker gene expression were significantly increased at 2 days post-differentiation (Figure 3D,E).

**Figure 4:**
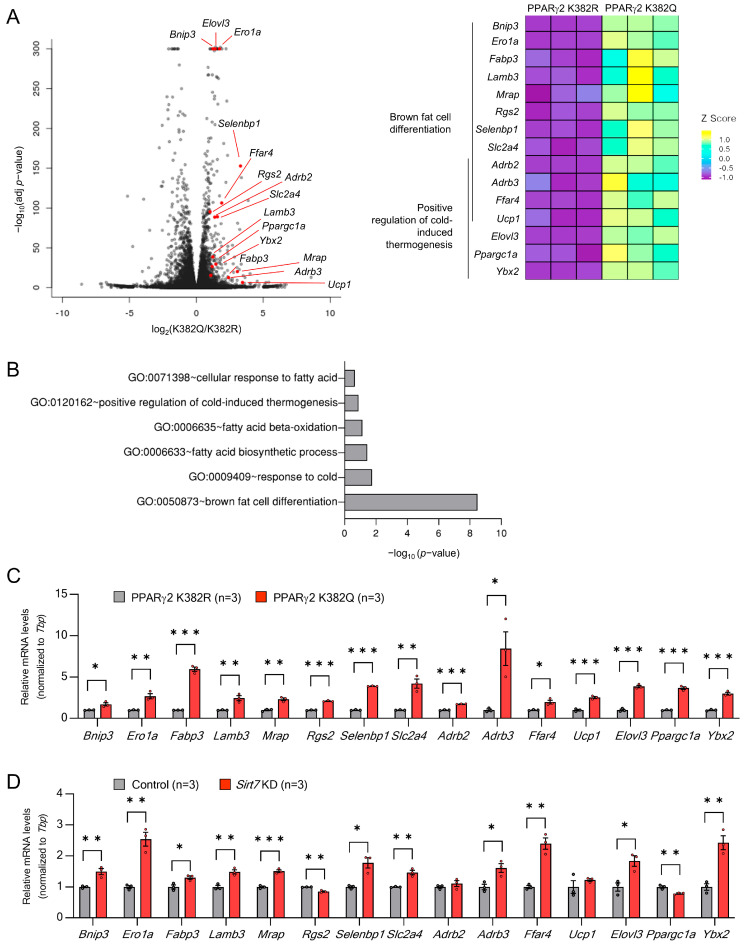
Acetylation of PPARγ2 at K382 enhances the expression of numerous genes involved in brown/beige adipocyte differentiation. (**A**,**B**) RNA-seq analysis of early differentiated (2 days post-differentiation) beige adipocytes from PPARγ2^K382Q^- and PPARγ2^K382R^-overexpressing mouse scWAT SVF cell lines. DEGs (adjusted *p*-value < 0.05, |Fold change| > 2) were used for GO analysis with DAVID. Volcano plot and heatmap of the DEGs (**A**) and biological processes in GO enrichment analysis (**B**) of the 476 upregulated genes in the PPARγ2^K382Q^-overexpressing cells compared with the PPARγ2^K382R^-overexpressing cells (FDR < 0.05). (**C**,**D**) Real-time qPCR analysis of brown/beige adipocyte differentiation marker genes in PPARγ2^K382Q^-overexpressing and PPARγ2^K382R^-overexpressing mouse scWAT SVF cell lines (**C**) and in control and *Sirt7* KD mouse scWAT SVF cell lines (**D**) at 48 h post-beige adipocyte differentiation. Data are presented as mean ± SEM of triplicates. * *p* < 0.05, ** *p* < 0.01, *** *p* < 0.001 with a two-tailed Student’s *t*-test.

We next conducted RNA-seq analysis of PPARγ2^K382Q^- and PPARγ2^K382R^-overexpressing cells at the early stages of beige adipocyte differentiation (2 days post-differentiation). Compared with PPARγ2^K382R^-overexpressing cells, 476 upregulated and 602 downregulated DEGs were detected in PPARγ2^K382Q^-overexpressing cells. Among the upregulated genes, *Ucp1*, *Ppargc1a*, *Elovl3* (ELOVL fatty acid elongase 3), and *Cidea* (cell death-inducing DNA fragmentation factor and alpha subunit-like effector A) were preferentially expressed in differentiating brown/beige adipocytes (Figure 4A). Enrichment analysis based on the GO biological process gene set showed that the 476 upregulated genes were enriched for terms related to brown fat cell differentiation and positive regulation of cold-induced thermogenesis (Figure 4B). qPCR analysis confirmed that the expression levels of a large number of genes involved in brown/beige adipocyte differentiation were significantly higher in PPARγ2^K382Q^-overexpressing cells (Figure 4C and Figure S3A). Consistently, the expressions of these genes were significantly increased in beige adipocytes from *Sirt7* KD mouse scWAT SVF cell lines (Figure 4D and Figure S3B). Taken together, these results indicate that SIRT7-mediated PPARγ2 deacetylation at K382 suppresses beige adipocyte differentiation.

### 3.4. Acetylation of PPARγ2 at K382 Represses the Expression of Type I and Type II Interferon-Stimulated Genes in Beige Adipocytes

**Figure 5:**
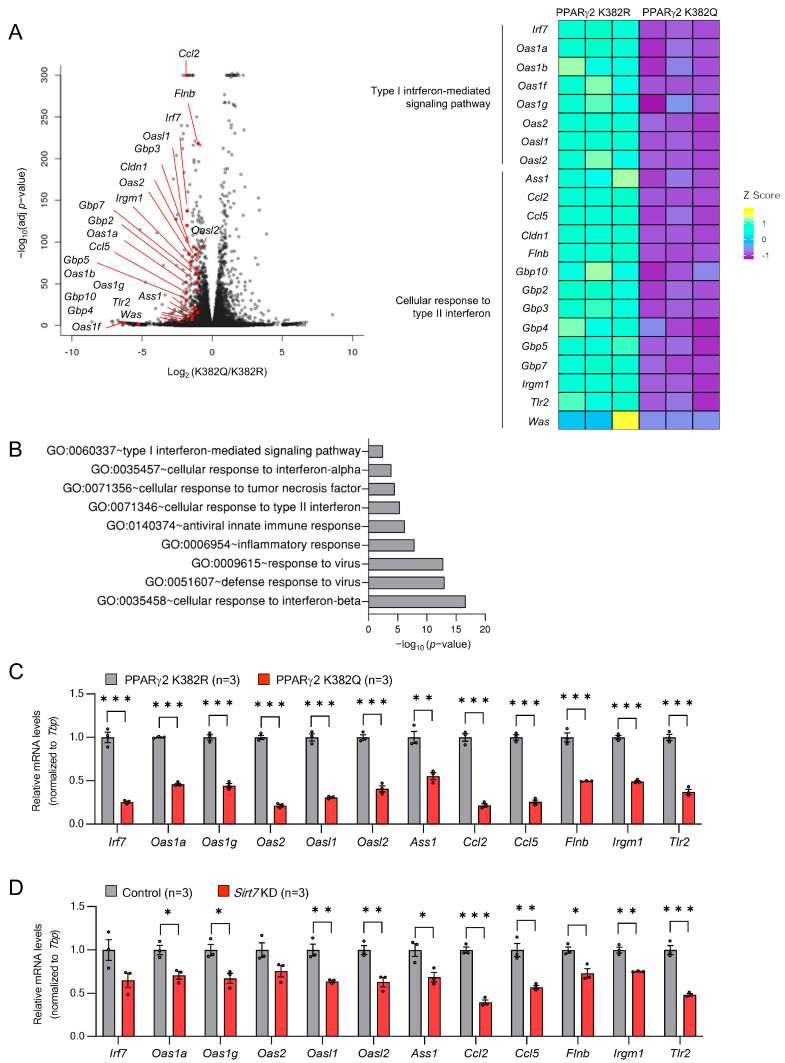
Acetylation of PPARγ2 at K382 represses the expression of type I and type II ISGs in beige adipocytes. (**A**,**B**) RNA-seq analysis of early differentiated (2 days post-differentiation) beige adipocytes from PPARγ2^K382Q^- and PPARγ2^K382R^-overexpressing mouse scWAT SVF cell lines. DEGs (adjusted *p*-value < 0.05, |Fold change| > 2) were used for GO analysis with DAVID. Volcano plot and heatmap of the DEGs (**A**) and biological processes in GO enrichment analysis (**B**) of the 602 downregulated genes in the PPARγ2^K382Q^-overexpressing cells compared with the PPARγ2^K382R^-overexpressing cells (FDR < 0.05). (**C**,**D**) Real-time qPCR analysis of type I and type II ISGs in PPARγ2^K382Q^-overexpressing and PPARγ2^K382R^-overexpressing mouse scWAT SVF cell lines (**C**) and in control and *Sirt7* KD mouse scWAT SVF cell lines (**D**) at 2 days post-beige adipocyte differentiation. Data are presented as mean ± SEM of triplicates. * *p* < 0.05, ** *p* < 0.01, *** *p* < 0.001 with a two-tailed Student’s *t*-test.

GO analysis of the downregulated genes in PPARγ2^K382Q^-overexpressing cells revealed significant enrichment for terms related to cellular response to interferon (IFN)-beta, cellular response to type II IFN, cellular response to IFN-alpha, and type I IFN-mediated signaling pathway (Figure 5A,B). PRDM16 was previously shown to repress the type I IFN response in preadipocytes derived from scWAT to promote mitochondrial and thermogenic programming [34]. Given that the expression of the *Prdm16* gene was upregulated in PPARγ2^K382Q^-overexpressing cells (Figure 3C), the acetylation of PPARγ2 at K382 may repress the expression of IFN-stimulated genes (ISGs) via *Prdm16* gene induction in beige adipocytes. Indeed, qPCR analysis confirmed that the expression levels of type I ISGs, such as *irf7*, *oas1a*, *oas1g*, *oas2*, *oasl1,* and *oasl2,* and of type II ISGs, such as *Ass1*, *ccl2, ccl5*, *flnb*, *irgm1,* and *tlr2*, were significantly downregulated in PPARγ2^K382Q^-overexpressing cells compared with PPARγ2^K382R^-overexpressing cells (Figure 5C). Consistently, the expression levels of these genes were substantially reduced in *Sirt7* KD mouse scWAT SVF cell lines at 48 h post-beige adipocyte differentiation (Figure 5D). These results indicate that SIRT7-mediated PPARγ2 deacetylation at K382 maintains the expression of ISGs in beige adipocytes.

### 3.5. Acetylation of PPARγ2 at K382 Strongly Recruits PRDM16 and PGC-1α and Activates PPARγ2 Transcriptional Activity in Beige Adipocytes

**Figure 6:**
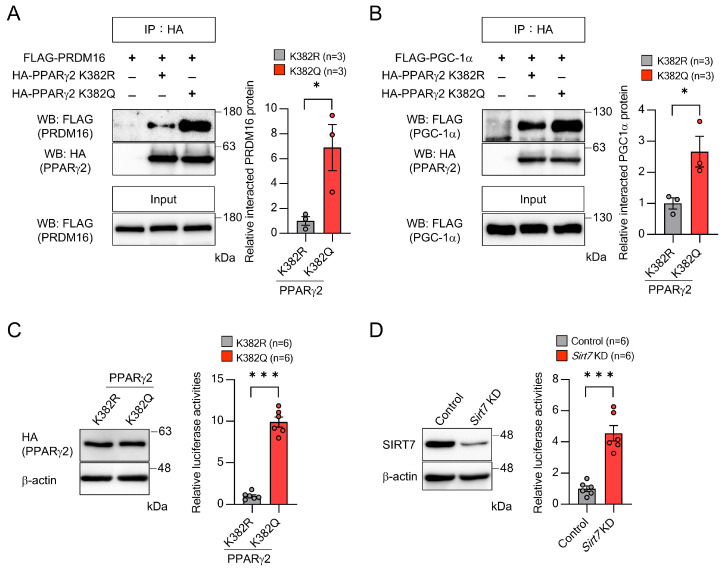
Acetylation of PPARγ2 at K382 strongly recruits PRDM16 and PGC-1α and activates PPARγ2 transcriptional activity in beige adipocytes. (**A**,**B**) Co-immunoprecipitation assay detecting the interaction of 3 × HA-PPARγ2^K382R^ or 3 × HA-PPARγ2^K382Q^ with FLAG-PRDM16 (**A**) and with PGC-1α (**B**) in HEK293T cells (left panel). The relative interaction of PPARγ2 with PRDM16 (**A**) and with PGC-1α (**B**) was quantified by normalization to immunoprecipitated PPARγ2 (right panel). (**C**,**D**) PPRE-driven luciferase reporter assay for assessing the transcriptional activities of PPARγ2. Differentiated PPARγ2^K382R^- or PPARγ2^K382Q^-overexpressing mouse scWAT SVF cell lines (**C**) and control or *Sirt7* KD mouse scWAT SVF cell lines (**D**) were transfected with pUC-3 × PPRE-tk-LUC and pRL-TK. Protein levels of PPARγ2 and SIRT7 were determined using Western blotting (left panel). Luciferase activity was determined after 24 h (right panel). WB, Western blotting; IP, immunoprecipitation. Data are presented as mean ± SEM of three independent experiments. * *p* < 0.05, *** *p* < 0.001 with a two-tailed Student’s *t*-test.

To elucidate the molecular mechanisms underlying the effect of the acetylation status of PPARγ2 at K382 on browning marker gene expression, we compared PPARγ2^K382R^ and PPARγ2^K382Q^ in terms of their binding to the transcriptional coactivators of browning PRDM16 and PGC-1α. The interaction of PRDM16 with PPARγ2^K382R^ or PPARγ2^K382Q^ was confirmed using a co-immunoprecipitation assay. As shown in Figure 6A, PPARγ2^K382Q^ showed a 7-fold increased interaction with PRDM16 compared with PPARγ2^K382R^. Similarly, the interaction with PGC-1α was increased more than 2-fold for PPARγ2^K382Q^ compared with PPARγ2^K382R^ (Figure 6B). These results suggest that PPARγ2^K382Q^ drives beige adipocyte differentiation by interacting with transcriptional coactivators of browning.

Next, to interpret the functional significance of this interaction, we examined the transcriptional activities of PPARγ2^K382R^ and PPARγ2^K382Q^ using a PPAR responsive element (PPRE)-driven luciferase reporter assay. PPARγ2^K382Q^ showed a 10-fold increase in transcriptional activity compared with PPARγ2^K382R^ in beige adipocytes from mouse scWAT SVF cell lines, even though the protein levels of PPARγ2^K382R^ and PPARγ2^K382Q^ were almost equal (Figure 6C). Consistently, in the same PPRE-driven luciferase reporter assay, the transcriptional activity of PPARγ2 was more than 4-fold higher in beige adipocytes from *Sirt7* KD mouse scWAT SVF cell lines compared with control cells (Figure 6D). Taken together, these results demonstrate that SIRT7 deficiency facilitates PPARγ2 acetylation at K382, thereby enhancing the interaction with PRDM16 and PGC-1α and promoting PPARγ2 transcriptional activity in differentiated beige adipocytes.

Conversely, in undifferentiated mouse scWAT SVF cell lines, the transcriptional activity of PPARγ2^K382Q^ was almost 3-fold lower than that of PPARγ2^K382R^ (Figure S4A), and SIRT7 deficiency decreased PPARγ2 transcriptional activity by more than 2-fold (Figure S4B). These findings suggest that, in terms of beige adipocyte precursors, the SIRT7-mediated deacetylation of PPARγ2 at K382 maintains PPARγ2 transcriptional activity.

### 3.6. SIRT7 Substantially Dissociates from PPARγ2 at the Onset of Beige Adipocyte Differentiation

**Figure 7:**
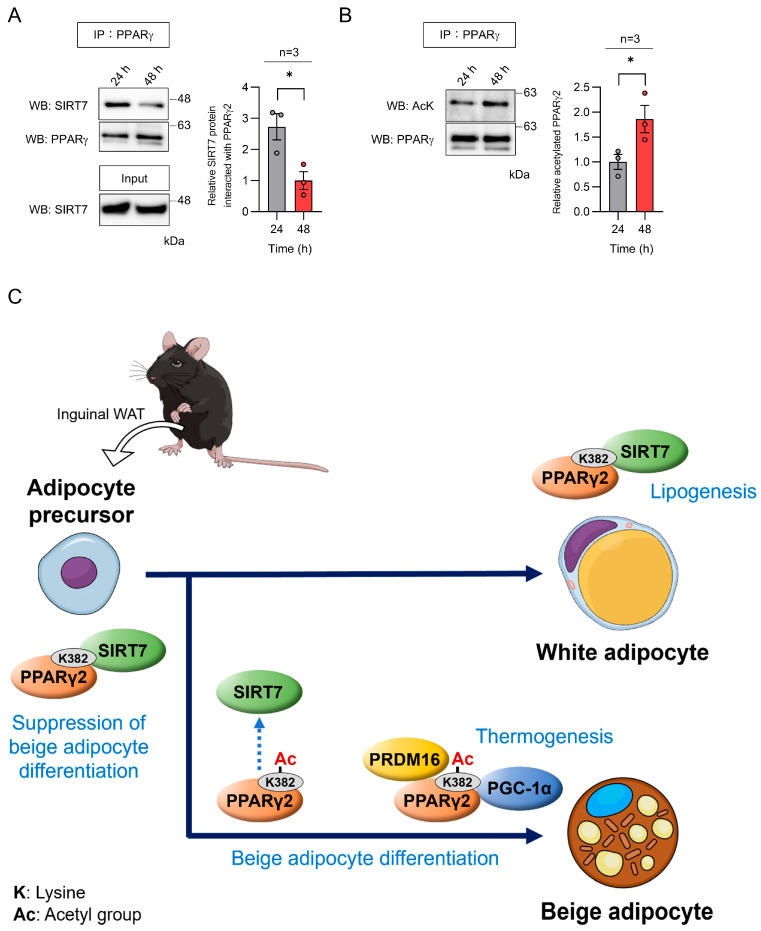
SIRT7 substantially dissociates from PPARγ2 at the onset of beige adipocyte differentiation. (**A**) Co-immunoprecipitation assay detecting the interaction between endogenous PPARγ and SIRT7 at 24 h and 48 h post-beige adipocyte differentiation in mouse scWAT SVF cell lines (left panel). The relative interaction of SIRT7 with PPARγ was quantified by normalization to immunoprecipitated PPARγ (right panel). (**B**) Acetylation assay for endogenous PPARγ at 24 h and 48 h post-beige adipocyte differentiation in mouse scWAT SVF cell lines (left panel). Quantification of the acetylated PPARγ relative to immunoprecipitated PPARγ (right panel). (**C**) Proposed model for the suppression of beige adipocyte differentiation via SIRT7-dependent deacetylation of PPARγ2. See Discussion for details. WB, Western blotting; IP, immunoprecipitation; AcK, acetylated lysine. Data are presented as mean ± SEM of three independent experiments. * *p* < 0.05 with a two-tailed Student’s *t*-test.

Given that SIRT7 suppresses the PPARγ2 transcriptional activity required for differentiated beige adipocytes, we wondered how endogenous SIRT7 regulates PPARγ2 transcriptional activity during beige differentiation. First, we examined the amount of SIRT7 at the onset of beige adipocyte differentiation by Western blotting. As shown in Figure 7A, SIRT7 protein levels were similar at 24 h and 48 h post-beige adipocyte differentiation in mouse scWAT SVF cell lines. Next, we used co-immunoprecipitation to investigate the interaction between endogenous PPARγ2 and SIRT7 at the onset of beige adipocyte differentiation. The interaction of PPARγ2 with SIRT7 was nearly 3-fold lower at 48 h post-differentiation compared with 24 h (Figure 7A). Consistently, the acetylation level of PPARγ2 was increased at 48 h post-differentiation compared with 24 h (Figure 7B). These results suggest that SIRT7 substantially dissociates from PPARγ2 at the onset of beige adipocyte differentiation to release the brake on differentiation.

## 4. Discussion

Both the activation and suppression mechanisms of AT browning are receiving considerable attention for the management of various metabolic disorders, including metabolic syndrome and cachexia [12]. We recently reported that browning-related gene expression was significantly elevated in inguinal WAT of norepinephrine-treated *Sirt7* AdKO mice compared with control mice [19]. The present results demonstrate that SIRT7 deficiency enhances beige adipocyte differentiation from mouse scWAT and human scBAT SVF cells. We also elucidated the molecular mechanism of the SIRT7-mediated suppression of AT browning, finding that SIRT7-mediated deacetylation of PPARγ2 at K382 interferes with the interaction with PRDM16 and PGC-1α. This suppresses the PPARγ2 transcriptional activity essential for beige adipocyte differentiation. Thus, this study establishes that SIRT7 is the only sirtuin family member to act as a critical thermogenic suppressor that puts the brake on AT browning.

PPARγ is a master regulator of adipogenesis and lipid metabolism that governs distinct downstream transcriptional targets in all three types of adipocytes [35]. In different cells or in response to different stimuli, PPARγ is subjected to various post-translational modifications and forms distinct complexes with cofactors and transcription partners to regulate specific transcriptional targets [36]. Based on these mechanisms, functionally selective PPARγ modulators have been developed that control the expression of a unique subset of PPARγ target genes without affecting the expression of classical white adipogenic genes [36]. We previously reported that SIRT7-mediated PPARγ2 deacetylation at K382 enhances lipogenesis in C3H10T1/2-derived white adipocytes but has no effect on white adipocyte differentiation [20]. In the present study, we demonstrated that SIRT7-mediated PPARγ2 deacetylation at K382 suppresses beige adipocyte differentiation. Thus, the acetylation status of PPARγ2 at K382 could produce distinct outcomes in different cellular contexts. Mechanistically, we elucidated that the deacetylation of PPARγ2 at K382 interferes with the interaction with PRDM16 and PGC-1α, which are essential for PPARγ2 transcriptional activation in beige adipocyte differentiation. A systematic search for cofactors or transcription factors interacting with PPARγ2^K382R^ in white adipocytes would help to elucidate the molecular mechanism of SIRT7-enhanced lipogenesis. We also found that the transcriptional activity of PPARγ2^K382R^ was significantly higher than that of PPARγ2^K382Q^ in adipocyte precursors. This finding supports the assumption that the SIRT7-mediated deacetylation of PPARγ2 at K382 is important in the maintenance of adipocyte precursors. Further studies of PPARγ2 downstream genes and PPARγ2^K382R^ transcriptional complexes in adipocyte precursors are necessary to uncover the role and precise mechanism of SIRT7 in adipose stem/precursor cells. In addition, we recently reported that SIRT7 suppresses energy expenditure in brown adipocytes in an insulin-like growth factor 2 mRNA-binding protein 2 (IGF2BP2/IMP2)-dependent manner, at least in part, without influencing brown adipocyte differentiation [19]. Accordingly, it is worth investigating whether the SIRT7-dependent deacetylation of PPARγ2 at K382 plays an important role in brown adipocytes. Given that PPARγ1 has the same ligand-binding domain as PPARγ2 and is expressed in a wide variety of tissues or cells, we expect the development of compounds that modulate the acetylation of K352 in PPARγ1 (K382 in PPARγ2), which may have benefits for various tissues affected by metabolic disorders.

SIRT1, 5, and 6 are major positive regulators of AT browning [37]. In particular, the underlying mechanism of SIRT1-enhanced AT browning has been elucidated: SIRT1 deacetylates PPARγ at K268 and K293, which recruits PRDM16, resulting in the selective induction of browning genes [16]. Nonetheless, SIRT1 does not constitutively induce browning genes in inguinal WAT at ambient temperature, and the mechanism by which SIRT1 is unable to deacetylate PPARγ at K268 and K293 in adipocyte precursors is still unclear. In the present study, we demonstrated that the SIRT7-regulated acetylation status of PPARγ2 at K382 is particularly important for beige adipocyte differentiation in the early onset of differentiation. We also observed that SIRT1 only weakly interacts with PPARγ2^K382R^ compared with PPARγ2^K382Q^ (Figure S5). Therefore, we propose the following model in which SIRT7 and SIRT1 cooperatively and sequentially regulate AT browning. In adipocyte precursors, SIRT7 deacetylates PPARγ2 at K382, limiting its interaction with PRDM16 and SIRT1, resulting in the repression of browning genes. Upon differentiation, PPARγ2 dissociates from SIRT7, thereby triggering the acetylation of PPARγ2 at K382. PPARγ2 acetylated at K382 recruits PRDM16 and PGC-1α and thus enhances beige adipocyte differentiation. Subsequently, SIRT1 deacetylates PPARγ at K268 and K293, which further recruits PRDM16, resulting in the full induction of browning genes. It will be interesting to determine whether SIRT1 contributes to the early onset of beige adipocyte differentiation, which has not yet been examined, and whether selective activators of SIRT1 further enhance beige adipocyte differentiation from *Sirt7* KD scWAT SVF cells.

Type I IFN signaling is critical for antiviral defense; however, low basal levels of type I IFN are also observed across various cell types in the absence of infection and are important for the activity of certain cell populations [38,39]. Kissig et al. reported that the ectopic activation of type I IFN signaling in brown and beige adipocyte precursors attenuated the expression of brown fat signature genes, such as *Ucp1* and *Cidea*, and caused profound mitochondrial dysfunction [34]. Furthermore, they revealed that PRDM16 limits the activation of IFN-induced genes by competing with IFN regulatory factor 1 (IRF1) for binding to IRF-E response elements at ISG promoters, thereby maintaining mitochondrial and thermogenic function in brown/beige adipocytes [34]. Kumari et al. reported that KO mice of IRF3, which is a key transcriptional regulator of type I IFN-dependent immune responses, have increased energy expenditure due to enhanced AT browning [40]. These reports suggest that type I IFN signaling serves as a critical brake on the thermogenic program in beige adipocyte precursors. In the present study, we demonstrated that SIRT7 deficiency and acetylation of PPARγ2 at K382 repress the expression of ISGs in beige adipocytes. Although it is suggested that reduced ISG expression in PPARγ2^K382Q^-overexpressing cells was mainly through *Prdm16* gene induction, other possibilities are not ruled out, because *Prdm16* gene induction was much higher in *Sirt7* KD cells than in PPARγ2^K382Q^-overexpressing cells while ISG suppression was lower. The expression of *Ppargc1a* was significantly activated in PPARγ2^K382Q^-overexpressing cells. A previous report [41] showed that PGC-1α suppresses the induction of IRF1 expression and phosphorylation of STAT1, an essential transcriptional activator of IRF1, suggesting that *Ppargc1a* gene induction in PPARγ2^K382Q^-overexpressing cells may contribute to the reduction in ISG expression. Taken together, our findings indicate that SIRT7-mediated PPARγ2 deacetylation at K382 suppresses the thermogenic programming of beige adipocyte precursors by maintaining the expression of ISGs, at least in part.

This study has several limitations. First, although we demonstrated that deacetylation of PPARγ2 at K382 suppresses beige adipocyte differentiation, we did not directly demonstrate SIRT7-mediated deacetylation of PPARγ2 at K382 during this process. Second, SIRT7 also functions as a lysine deacylase, mediating depropionylation and demyristoylation [29]; however, the contributions of these deacylase activities to beige adipocyte differentiation remain unclear. Third, we were unable to determine the reasons for the differences in lipid droplet accumulation between *Sirt7* KD mouse scWAT cells and SIRT7 KD human scBAT cells. Fourth, the sample size of in vitro experiments (Figure 2, Figure 3, Figure 4 and Figure 5) was small, although some experiments were independently replicated using cells derived from different KD lines or passages. Nevertheless, this study has yielded important results. Larger, but otherwise similar, studies are required to reconfirm these findings.

## 5. Conclusions

In conclusion, AT browning has emerged as a promising therapeutic target for decreasing body weight and improving insulin sensitivity in obesity and diabetes. However, in individuals with burns or cancer, AT browning exhibits adverse clinical outcomes related to cachexia, hepatic steatosis, and muscle catabolism [12]. Therefore, the development of therapeutic strategies for metabolic diseases requires approaches that precisely regulate both the induction and suppression of AT browning. This study suggests that SIRT7 acts as a brake in AT browning, and that it may be a potential target for the treatment of metabolic disorders, including hypermetabolic conditions.

## Data Availability

RNA-seq datasets generated during this study are available at Gene Expression Omnibus (GEO). Accession number: GSE317859. The data that support the findings of this study are available from the corresponding author upon reasonable request.

## Supplementary Materials

The following supporting information can be downloaded at: https://www.mdpi.com/article/10.3390/cells15111028/s1, Figure S1: Real-time qPCR analysis of beige adipocytes from mouse scWAT SVF cell lines and brown adipocytes from mouse BAT SVF cell lines; Figure S2: Oil red O staining of fully differentiated beige adipocytes from control and *Sirt7* KD human scBAT SVF cell lines; Figure S3: Acetylation of PPARγ2 at K382 enhances the expression of numerous genes involved in brown/beige adipocyte differentiation; Figure S4: SIRT7-mediated deacetylation of PPARγ2 at K382 maintains PPARγ2 transcriptional activity in beige adipocyte precursors; Figure S5: SIRT1 only weakly interacts with PPARγ2^K382R^ compared with PPARγ2^K382Q^; Table S1: List of primers used in the present study.
