# Supplementary material for: SIRT7 Inhibits Adipose Tissue Browning Through Deacetylation of PPARγ2 at K382
Source: Cells. 2026 Jun 3;15(11):1028. doi: 10.3390/cells15111028 (PMC13256863; doi:10.3390/cells15111028)
Supplement: Supplementary file 1 [file cells-15-01028-s001.zip › cells-4311397-supplementary.pdf]

Supplementary materials

SIRT7 inhibits adipose tissue browning through deacetylation of PPARγ2 at K382

Avizit Das [1], Tatsuya Yoshizawa [1,2,*], Daisuke Yamada [2], Tomonori Tsuyama [3], Yoshifumi Sato [1], Tomoya Mizumoto [1], Takeshi Yoneshiro [4,5], Shingo Kajimura [6], Kazuya Yamagata [1,3,**]

[1]Department of Medical Biochemistry, Faculty of Life Sciences, Kumamoto University, Kumamoto 860-8556, Japan.
[2]Cell Biology, Graduate School of Medical Science, Kyoto Prefectural University of Medicine, Kyoto 606-0823, Japan.
[3]Center for Metabolic Regulation of Healthy Aging, Faculty of Life Sciences, Kumamoto University, Kumamoto 860-8556, Japan.
[4]Division of Molecular Physiology and Metabolism, Tohoku University Graduate School of Medicine, Sendai, Miyagi 980-8575, Japan
[5]Division of Metabolic Medicine, Research Center for Advanced Science and Technology (RCAST), The University of Tokyo, Tokyo 153-8904, Japan
[6]Beth Israel Deaconess Medical Center, Harvard Medical School, and Howard Hughes Medical Institute, Boston, MA 02215, USA.
*Corresponding author. E-mail: yoshizaw@koto.kpu-m.ac.jp (T. Yoshizawa).
**Corresponding author. E-mail: k-yamaga@kumamoto-u.ac.jp (K. Yamagata).

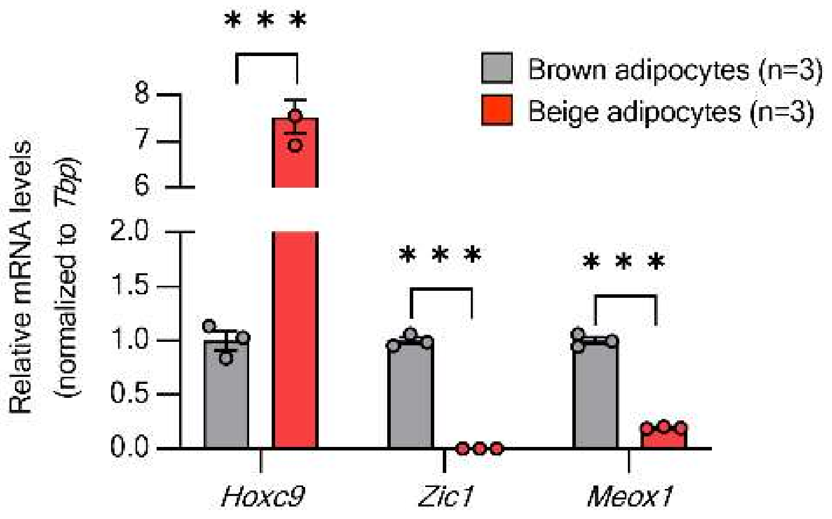

Figure S1: Real-time qPCR analysis of beige adipocytes from mouse scWAT SVF cell lines and brown adipocytes from mouse BAT SVF cell lines. To confirm the appropriate differentiation to beige or brown adipocytes, the gene expressions of white/beige (*Hoxc9*) and brown (*Zic1* and *Meox1*) adipocyte markers were examined. Data are presented as mean ± SEM of triplicates. *$p < 0.05$, **$p < 0.01$, ***$p < 0.001$ with a two-tailed Student's *t*-test.

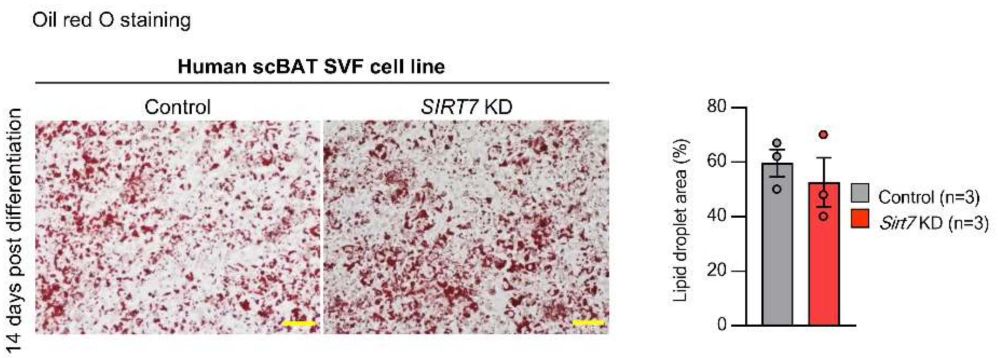

Figure S2: Oil red O staining of fully differentiated beige adipocytes from control and *Sirt7* KD human scBAT SVF cell lines. Oil red O staining of 14 days post beige differentiated control and *Sirt7* KD human scBAT SVF cell lines (left panel, scale bar = 100 μm), and quantification of the stained oil red O (right panel). Data are presented as mean ± SEM of triplicates. *$p < 0.05$, **$p < 0.01$, ***$p < 0.001$ by two-tailed Student's t-test.

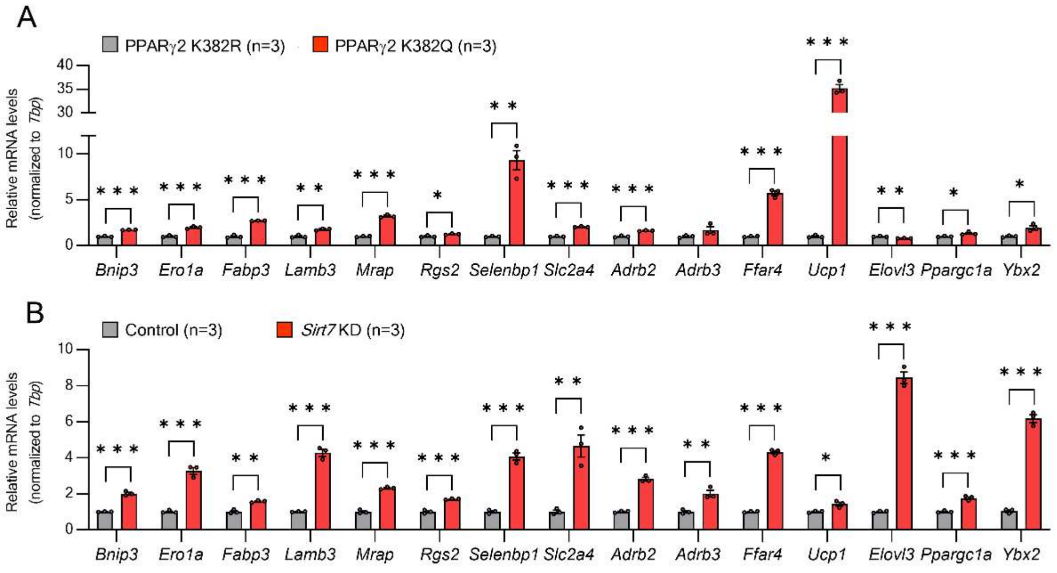

Figure S3: Acetylation of PPARγ2 at K382 enhances the expression of numerous genes involved in brown/beige adipocyte differentiation. A, B Real-time qPCR analysis of brown/beige adipocyte differentiation marker genes in PPARγ2^{K382Q}-overexpressing and PPARγ2^{K382R}-overexpressing mouse scWAT SVF cell lines (**A**) and in control and *Sirt7* KD mouse scWAT SVF cell lines (**B**) at full beige adipocyte differentiation (7 days post-differentiation). Data are presented as mean ± SEM of triplicates. **p* < 0.05, ***p* < 0.01, ****p* < 0.001 with a two-tailed Student's *t*-test.

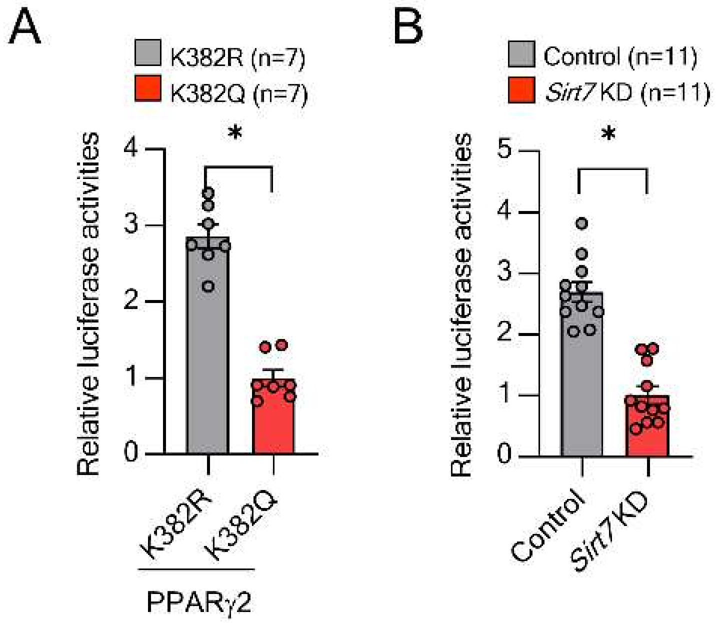

Figure S4: SIRT7-mediated deacetylation of PPARγ2 at K382 maintains PPARγ2 transcriptional activity in beige adipocyte precursors. A, B PPRE-driven luciferase reporter assay for assessing the transcriptional activities of PPARγ2. Undifferentiated PPARγ2^{K382R}- or PPARγ2^{K382Q}-overexpressing mouse scWAT SVF cell lines (**A**) and control or *Sirt7* KD mouse scWAT SVF cell lines (**B**) were transfected with pUC-3×PPRE-tk-LUC and pRL-TK. Luciferase activity was determined after 24 h. Data are presented as mean ± SEM of triplicates. *$p < 0.05$, **$p < 0.01$, ***$p < 0.001$ with a two-tailed Student's t-test.

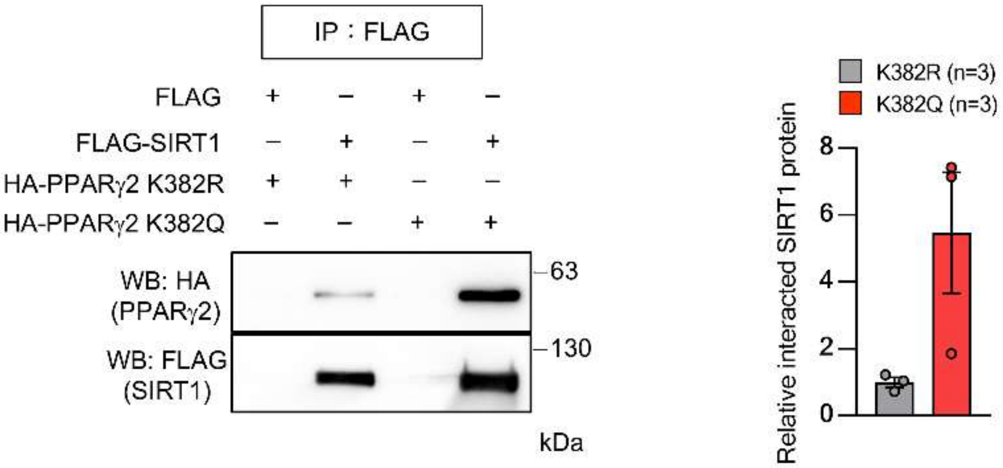

Figure S5: SIRT1 only weakly interacts with PPARγ2^{K382R} compared with PPARγ2^{K382Q}. Co-immunoprecipitation assay detecting the interaction of 3×HA-PPARγ2^{K382R} or 3×HA-PPARγ2^{K382Q} with FLAG-SIRT1 in HEK293T cells (left panel). The relative interaction of PPARγ2 with SIRT1 was quantified by normalization to immunoprecipitated PPARγ2 (right panel). Data are presented as mean ± SEM of three independent experiments. *$p < 0.05$, **$p < 0.01$, ***$p < 0.001$ with a two-tailed Student's *t*-test.

Table S1: List of primers used in the present study

	Genes	Forward primer	Reverse primer
Mouse	*Rpl19*	AAGCCTGTGACTGTCCATTC	CTTCTTGGATTCCCGGTATC
	Tbp	CCCCTTGTACCCTTCACCAAT	GAAGCTGCGGTACAATTCCA
	Pecam1	ATGGAAAGCCTGCCATCATG	TCCTTGTTGTTCAGCATCAC
	mt-Nd1	CCTATCACCCTTGCCATCAT	GAGGCTGTTGCTTGTGTGAC
	Hoxc9	ACTCGCTCATCTCTCACGACA	CTACAGGACGGAAAATCGCTACAGT
	Zic1	AACCTCAAGATCCACAAAAGGA	CCTCGAACTCGCACTTGAA
	Meox1	CCGGAGATATGAGATTGCAGTCAA	CCCTTCACACGTTTCCACTTCAT
	Ucp1	GGCAACAAGAGCTGACAGTAAAT	GGCCCTTGTAAACAACAAAATAC
	Prdm16	GGCGAGGAAGCTAGCCAAA	GGTCTCCTCCTCGGCACTCT
	Ppargc1a	GAAATCCGAGCGGAGCTGAA	GAATAGGGCTGCGTGCCATC
	Cidea	CTTGGGGGTGGTACCCAGTG	ATCCACGCAGTTCCCACACA
	Elovl3	TTGGGGATAGGGGGTGTGTG	TCTCCCCTCCCCTCCAAGTC
	Fgf21	GCCATTCACTTTGCCTGAGC	ATCCATTCCATCAGGGCTGC
	Cited1	CCAACCTTGGAGTGAAGGATCGCA	GGCAGTAGGAGAGCCTATTGGAGATGT
	Pat2	GTGCCAAGAAGCTGCAGAG	TGTTGCCTTTGACCAGATGA
	P2rx5	CTGCAGCTCACCATCCTGT	CACTCTGCAGGGAAGTGTCA
	Selenbp1	GCTGATACTGCCTGGTCTCA	AGTGGCTGGTGTGCAAAC
	Rgs2	AGGAAGCTCCCAAAGAGATAAACAT	CCGTGGTGATCTGTGGCTTTTTAC
	Lamb3	ACAGAGTTGAAGGACCGGCTGG	TCGAAGCAGCTCTGACTCCATGTC
	Ero1a	GCTGCTTCTGCCAGGTTAGTGGTTA	GGGTTTGACGGCACAGTCTCTTC
	Bnip3	CTGCAGGGCTCCTGGGTAGA	TCATGCTGGGCATCCAACAG
	Fabp3	TCTGCCAACTGGCCACCCCT	TGACCTTGGAGCACCCTTTGGA
	Mrap	TGTGGTTGAGCCTGGCTACCTT	GGAGGTTGAAGCTGTGAGTCCA
	Slc2a4	ACCCCTCATTCCCCCTGTGT	ACCCTCCTGCAGACCCCTTC
	Ffar4	TGGCCATCCCTTTTCTTCTGG	AAATGGCTCCCTTCTCTGGAA
	Adrb2	GAGCGACTACAAACCGTCACCA	TGGAAGTCCAGAACTCGCACCA
	Adrb3	CGCTACCTAGCTGTCACCAACCC	GGGCATGTTGGAGGCAAAGGAAC
	Ybx2	AGCCCATAGAGGGCTCTGATG	AGGCCGGGAGCCATCAGT
	Oasl1	CCAGGAAGAAGCCAAGCACCATC	AGGTTACTGAGCCCAAGGTCCATC
	Oas1a	CCAAGGTGGTGAAGGGTGG	ACCACCAGGTCAGCGTCTGA

	Oas1g	GTTGGCTGAAGAGGCTGATGT	AGTTCTCCTCCACCTGCTCAA
	Oas2	CCGGGCCAGTGCACAAGTTAG	CGATGGCACCGAGGACACC
	Oasl2	GGATGCCTGGGAGAGAATCGA	CTCGCCTGCTCTTCGAAACTG
	Irf7	GCCAGGAGCAAGACCGTGTT	TGCCCCACCACTGCCTGTA
	Ass1	AAAACTCAGGACCCTGCCAAAGC	TGCGGGTTGTGCCATCTTTGATG
	Flnb	AGGGCAAGAACAGCGTGGAG	GCTTCCAGCAGCCTTGGTGT
	Tlr2	AGTGGCCAGAAAAGATGCGCTTC	TTGCAGCCGAGGCAAGAACAAAG
	Irgm1	TCTGGCAATGGCATGTCATCTT	AGTACTCAGTCCGCGTCTTCGT
	Ccl2	AAGAGGATCACCAGCAGCAGG	GTATGTCTGGACCCATTCCTTCTTGG
	Ccl5	ACACCACTCCCTGCTGCTTTG	TTGGCACACACTTGGCGGTTC
Human	*Tbp*	CACGCCAGCTTCGGAGAGTT	TGGCTCCTGTGCACACCATT
	Ucp1	ACAAAAGTGTGCCCAACTGTGCAATG	CGTTCCAGGATCCAAGTCGCAAGAAGG
	Prdm16	ACGAGGCCCCTGTCTACATTCCTG	GCTCCCATCCGAAGTCTGTCTCCT
	Cidea	CGGGACTCAAGGGCCTGCTGA	TATCCACACGTGAACCTGCCCTTGG
	Ppargc1a	AGAGATTCTGTGTCACCACCCAAATCCTT	ACGACCTGTGTCGAGAAAAGGACCTTG
	Cited1	CAACCTTGCGGTGAAAGATCG	TAGGAGAGCCTATTGGAGATCCC
	Fgf21	GCCTTGAAGCCGGGAGTTATT	AGTGGAGCGATCCATACAGGG
	Pat2	GCCTGCCACTGTATGCACATC	TTCTAGTCCATGCATCACCGTGT
	P2rx5	CCTTCACCAACACCTCGGATCT	CACAATCAGGTTGGTGACCACAA
